# Structural and Functional Connectivity of the Anterior Cingulate Cortex in Patients With Borderline Personality Disorder

**DOI:** 10.3389/fnins.2019.00971

**Published:** 2019-09-13

**Authors:** Xiaoxia Lei, Mingtian Zhong, Bowen Zhang, Huihui Yang, Wanrong Peng, Qian Liu, Yu Zhang, Shuqiao Yao, Changlian Tan, Jinyao Yi

**Affiliations:** ^1^Medical Psychological Center, The Second Xiangya Hospital, Central South University, Changsha, China; ^2^Center for Studies of Psychological Application, School of Psychology, South China Normal University, Guangzhou, China; ^3^Medical Psychological Institute, Central South University, Changsha, China; ^4^Department of Radiology, The Second Xiangya Hospital, Central South University, Changsha, China

**Keywords:** borderline personality disorder, emotion, anterior cingulate cortex, resting state functional connectivity, probabilistic fiber tracking

## Abstract

**Background:**

Emerging evidences supported the hypothesis that emotional dysregulation results from aberrant connectivity within the fronto-limbic neural networks in patients with borderline personality disorder (BPD). Considering its important role in emotional regulation, the anterior cingulate cortex (ACC) has not yet been fully explored in BPD patients. Therefore, using the seed-based resting state functional connectivity (rsFC) and probabilistic fiber tracking, we aimed to explore the alterations of functional and structural connectivity (SC) of the ACC in patients with BPD.

**Methods:**

A cohort of 50 unmedicated, young BPD patients and 54 sex-, age-, and education-matched healthy controls (HCs) completed psychological tests and underwent rs-fMRI and diffuse tensor imaging (DTI) scanning. Rs-FC analysis and probabilistic fiber tracking were used to plot SC and FC of the ACC.

**Results:**

With the left ACC selected as a seed, BPD patients exhibited increased rsFC and abnormal SC with the right middle frontal gyrus (MFG), and decreased rsFC with the left middle temporal gyrus (MTG), compared with HCs. Additionally, negative cognitive emotion regulation and depressive symptoms both correlated negatively with the rsFC of the left ACC in BPD patients.

**Conclusion:**

Abnormal SC and FC of the ACC underlie the deficient emotional regulation circuitry in BPD patients. Such alterations may be important biomarkers of BPD and thus could point to potential BPD treatment targets.

## Introduction

Borderline personality disorder is a common psychiatric disorder, characterized by a pervasive pattern of emotional lability, impulsivity, interpersonal difficulties, identity disturbances, and disturbed cognition (Leichsenring et al., 2011). Emotional dysregulation, which is considered a core pathological feature of BPD (Gratz and Roemer, 2004; Dsm-5, 2013), has been attributed to weakened inhibitory effects of the prefrontal cortex (PFC) on hyperactive limbic brain regions in response to emotional stimuli (Krause-Utz et al., 2014b; Schulze et al., 2016).

The PFC involvement in emotion regulation has been supported by neuroimaging studies in which control of emotion-related behavior was related to the activation of several frontal regions, including the ACC, dorsolateral PFC, and ventral medial PFC (Koenigsberg et al., 2009; Krause-Utz et al., 2012; Kamphausen et al., 2013; Ruocco et al., 2013). For example, activation of the ACC and other prefrontal structures (e.g., dorsolateral PFC and medial or orbital PFC) has been found to be consistently decreased during emotional processing in BPD patients compared with HCs (Silbersweig et al., 2007; Kraus et al., 2010; Smoski et al., 2011). Importantly, decreased PFC activation was reported to be more pronounced in response to negative emotional stimuli than to neutral stimuli (Schulze et al., 2016).

Apart from aberrant functional alterations of the ACC and other frontal structures, there also have been reported structural and biochemical aberration in these frontal lobes in BPD studies. Decreased gray matter volume (Hazlett et al., 2005; Minzenberg et al., 2008; Schulze et al., 2016) and thickness (Rüsch et al., 2007) in ACC, and reduced gray matter volumes in the orbitofrontal cortex and dorsolateral PFC (Chanen et al., 2008; Soloff et al., 2012), have been reported in patients with BPD, compared with HCs. In addition, BPD patients have increased glutamate and *N*-acetylaspartate concentrations in the left ACC (Hoerst et al., 2010; Rüsch et al., 2010a) and decreased *N*-acetylaspartate concentrations in the dorsolateral PFC (Tebartz van Elst et al., 2001).

Previous neuroimaging studies on BPD suggested that abnormal functional activity in frontal regions might be interrelated rather than independent (New et al., 2007; Niedtfeld et al., 2012; Kamphausen et al., 2013; Krause-Utz et al., 2014a). The frontal lobes and the ACC in particular played important roles in emotional cognitive control, and their deactivation was related strongly to negative emotional processing in BPD patients (Bush et al., 2000). Anatomically, the ACC has rich structural and functional connections with other regions in the frontal lobes. For example, the subgenual cingulate gyrus (part of the ventral medial cingulate gyrus) receives afferent projections from the orbital cortex and has a close relationship with part of the orbital–frontal cortex (Rüsch et al., 2010b). These connections help to integrate sensory information, coordinate autonomic nervous responses, and regulate emotion and behavior (Kringelbach, 2005). However, few studies have investigated alterations in SC or FC of emotion-related brain regions in BPD, especially the ACC. In one study investigating interhemispheric SC in BPD with a novel fiber-tracking approach, impaired connectivity between the left and right ACCs was observed (Rüsch et al., 2010b). Further investigations focusing on the ACC and other prefrontal areas involved in emotion regulation should be performed to confirm whether there is impaired interhemispheric connectivity in these patients (Rüsch et al., 2007, 2010b).

In this study, we used a multimodal approach combining rsFC and probabilistic fiber tracking with the bilateral ACC as seeds, to explore SC and FC of the ACC with other frontal regions, and how they relate to BPD-relevant psychological variables, namely negative affect intensity, negative cognitive emotion regulation strategies, depression, and anxiety. To exclude the influence of age, drug use, and comorbidity, we used a young BPD group with no antipsychotic drug use and no comorbidities. The severity of depression and anxiety symptoms was controlled as covariates to limit the influence of possible confounding effects.

## Materials and Methods

### Subjects

Patients diagnosed with BPD were recruited from an outpatient clinic affiliated with the Second Xiangya Hospital of Central South University in Changsha, Hunan province, China. HCs were recruited by advertisements from the surrounding communities. BPD diagnostic assessments were performed by two well-qualified psychiatrists using the structured clinical interview for axis II disorders (SCID II, First and Gibbon, 2004) of the Diagnostic and Statistical Manual of Mental Disorders, Fourth Edition (DSM-IV). Each patient received the Structured Clinical Interview for DSM-IV Axis I disorders (SCID I, First and Gibbon, 2004) to exclude past or current Axis I diagnoses (e.g., major depressive disorder, bipolar disorder, schizophrenia, delusional disorder, and schizo-affective disorder). The exclusion criteria for HCs were past or current history of any DSM-IV axis I or axis II disorder, a history of epilepsy or serious trauma, current medical problems, and family history of psychiatric disorders among first-degree relatives.

The study cohort included 50 patients with BPD and 54 age-, sex-, and education-matched HCs. All participants completed the rs-fMRI and T1 scanning; and of the 104 participants, 46 BPD patients and 44 HCs were scanned with DTI sequences. This study was approved by the ethics committee of the Central South University, and each participant signed an informed consent form at enrollment.

### Scales

Prior to neuroimaging, all participants underwent several psychometric assessments, including the Personality Diagnostic Questionnaire (PDQ-4+), Short Affect Intensity Scale (SAIS), Cognitive Emotion Regulation Questionnaire (CERQ), Center for Epidemiologic Studies Depression Scale (CES-D), and State-Trait Anxiety Inventory (STAI). The PDQ-4+, a self-rated personality disorder questionnaire, includes 12 sub-scales corresponding to 12 kinds of personality disorders. The borderline sub-scale contains nine items adopting 0–1 scoring method (i.e., Yes or No), and the total score ranges from 0 to 9, which ≥5 indicates a screened positive BPD (Hyler et al., 1992). The SAIS was used to assess affect intensity, which includes three dimensions (positive intensity, negative affectivity, and serenity; Geuens and De Pelsmacker, 2002; Zhong et al., 2010). Only the negative affectivity score (range, 1–6) assessing negative emotional reactions (e.g., nervousness, worry, sadness, and fear) was used in the current study. The CERQ is used to measure cognitive emotion regulation strategies with positive and negative subscales (Zhu et al., 2008). In the current study, only the negative subscale was used to assess subjects’ negative emotion regulation strategies (e.g., self-blame, rumination, catastrophization, and blaming others). The STAI and the CES-D were used to assess anxiety and depression levels, respectively (Shek, 1993; Xiao et al., 2016).

### MRI Parameters

Magnetic resonance imaging data were collected on a 3.0-T Philips Ingenia, in which foam pads were used to position and immobilize each subject’s head within the coil. High-resolution anatomical T1-weighted scans were obtained with the following parameters: repetition time (TR) = 7.44 ms; echo time (TE) = 3.46 ms; flip angle = 8°; sagittal slices = 301; slice thickness = 1.2 mm; slice spacing = 0.6 mm; matrix size = 240 × 240; volume = 1; and voxel size = 1 × 1 × 1 mm^3^.

Resting state functional magnetic resonance imaging images were obtained with following parameters: TR = 2000 ms; TE = 30 ms; flip angle = 90°; slices = 36; slice thickness = 4 mm; slice spacing = 4 mm; matrix size = 128 × 128; volumes = 200; volume interval = 2 s; and voxel size = 2 × 2 × 2 mm^3^. While being scanned, all participants were asked to relax, keep their eyes closed, and stay awake.

Diffuse tensor imaging images were acquired with the following parameters: TR = 6176 ms; TE = 79 ms; flip angle = 90°; slices = 60; slice thickness = 2.5 mm; slice spacing = 2.5 mm; and matrix size = 144 × 144, voxel size = 2 × 2 × 2 mm^3^. Diffusion-sensitizing gradient encoding was applied in 32 directions with a diffusion-weighted factor of *b* = 700 s/mm^2^ and two b0 (*b* = 0) images. Images were acquired parallel to the anterior and posterior commissure.

### Imaging Data Processing

#### Rs-fMRI Processing

Resting state functional magnetic resonance imaging data were analyzed in DPARSF (Data Processing Assistant for rs-fMRI software^1^; Yan and Zang, 2010), based on Statistical Parametric Mapping 12 (SPM12^2^) in MATLAB (Release 2017, The MathWorks, Inc., Natick, MA, United States). Data processing included the following steps: convert DICOM images to NIfTI files; remove the first 10 time points to minimize the influence of instability in the initial signals; slice timing with the 18th slice as the reference; spatial realignment for head motion correction (subjects with head motion >2.0 mm or >2.0° were excluded); register each subject’s fMRI images to their segmented high-resolution T1-weighted anatomical images; regress nuisance variables, including white matter (WM) and cerebral spinal fluid signals; normalize fMRI images to standard Montreal Neurological Institute (MNI) templates with a resolution of 3 × 3 × 3 mm^3^; smooth with a 4-mm full-width-half-maximum Gaussian kernel; linear detrend to discard physiological noise and drift from scanner instabilities and head motion; band-pass filtering (0.01–0.08 Hz); and calculate rsFC with the bilateral ACC selected as seeds, which were made by the Anatomical Automatic Labeling 90 regions (AAL90) templates to quantify the relationship between the seeds and other brain regions.

#### DTI Processing

Diffuse tensor imaging data were processed on the FMRIB’s Diffusion Toolbox-FDT v2.0 toolbox from FSL-FMRIB Software Library, FMRIB, Oxford, United Kingdom^3^ (Jenkinson et al., 2012). DTI data processing included the following steps: correct for eddy current distortion and head motion; fit a diffusion tensor model using the DTI fit in the FMRIB Diffusion Toolbox generating individual FA and MD; calculate within-voxel probability density functions of the principal diffusion direction using FSL’s BEDPOSTX tool using Markov Chain Monte Carlo sampling, which also accounts for the possibility of crossing fibers within a voxel (Woolrich et al., 2009); conduct probabilistic fiber tracking with PROBTRACKX implemented in FSL (Behrens et al., 2007), which repeatedly samples the distribution at each voxel to produce “streamlines” that connect voxels between selected seed regions (5000 streamline samples, 0.5 mm step length, curvature threshold = 0.2).

#### Probabilistic Fiber Tracking Analysis

Probabilistic fiber tracking analysis was based on the rsFC results and mainly investigated SC between which had abnormal functional connectivities in rs-fMRI analysis, i.e., the left ACC was selected as the seed, and the right MFG and left MTG as the targets. All selections were made by the AAL90 templates in FSL. A single image (fdt_paths) per participant was generated to visualize WM tracts connecting seed and target regions after data processing. The total number of seed-to-target WM tracts (i.e., waytotal) was also generated to calculate the connective probability or tract strength, which defined as the number of tracts from the seed, divided by the number of tracts that reach the target (van den Bos et al., 2014; Yuan et al., 2018).

After probabilistic tracking, generated fdt_paths images were further thresholded based on the individual maximum connectivity value within a tract. The maximum connectivity value was obtained with fslstats and voxels, which had values of >5% of the maximum connectivity value were kept in the analysis (Bennett et al., 2011; Hirsiger et al., 2016; Yuan et al., 2017). A common tract was created from tracts existed in ≥75% of the participants. Then, we extracted individual FA and MD of the WM tracts by de-projecting the common tracts to individual diffusion images (Ethofer et al., 2012; Gschwind et al., 2012).

### Statistical Analysis

Statistical analyses were conducted in SPSS 20.0 (SPSS Inc., Chicago, IL, United States) and the SPM12 toolbox of MATLAB. To compare group differences in demographic and psychological variables, Chi-squared tests and independent-sample *t*-tests were conducted. To investigate the significantly altered rsFC, two sample *t*-tests were performed in SPM12 with depression and anxiety scores as covariates. All resulting maps were family-wise error (FWE) corrected for the whole brain. To explore potential group differences in SC (i.e., probability) and integrity (i.e., FA and MD), analysis of covariance (ANCOVA) was performed with age, depression, and anxiety levels controlled. Pearson’s correlations were conducted to investigate potential associations between emotional characteristics (i.e., psychometric scores including SAIS_N, CERQ_N, CES-D, TAI, and SAI) and neuroimaging indices (i.e., rsFC values, tract probability, mean FA, and MD) in the BPD group. A threshold of *P* < 0.05 was considered significant.

## Results

### Demographic and Psychological Characteristics

Age, sex, and education level did not differ significantly between the BPD and HC groups. The BPD group had significantly higher SAIS negative affectivity, CERQ negative subscale, CES-D, and STAI scores than the HC group (Table 1).

**TABLE 1:** Inter-group comparisons of demographic and psychological characteristics.

**Characteristic**	**BPD group**	**HC group**	***t*/χ^2^**	***P***
	**(*N* = 50)**	**(*N* = 54)**		
Age	25.332.93	24.831.37	1.11	0.27
Sex ratio, male/female^a^	25/25	21/33	1.3	0.25
Education (y)	15.821.05	16.211.28	–1.68	0.09
PDQ-4+_borderline	3.891.98	0.760.97	9.42	<0.01
SAIS_N	4.290.82	3.490.65	5.53	<0.01
CERQ_N	40.705.66	34.785.04	8.07	<0.01
CES-D	37.3210.20	28.705.67	4.42	<0.01
SAI	37.7210.09	29.366.07	4.35	<0.01
TAI	43.578.71	32.785.22	6.21	<0.01

*PDQ-4+, Personality Diagnostic Questionnaire; SAIS_N, negative affectivity score of Short Affect Intensity Scale; CERQ_N, negative subscale of Cognitive Emotion Regulation Questionnaire; CES-D, Center for Epidemiologic Studies Depression Scale; SAI, State Anxiety Inventory; TAI, Trait Anxiety Inventory. ^*a*^Chi-square test; all other *t*-tests.*

### RsFC

**FIGURE 1:**
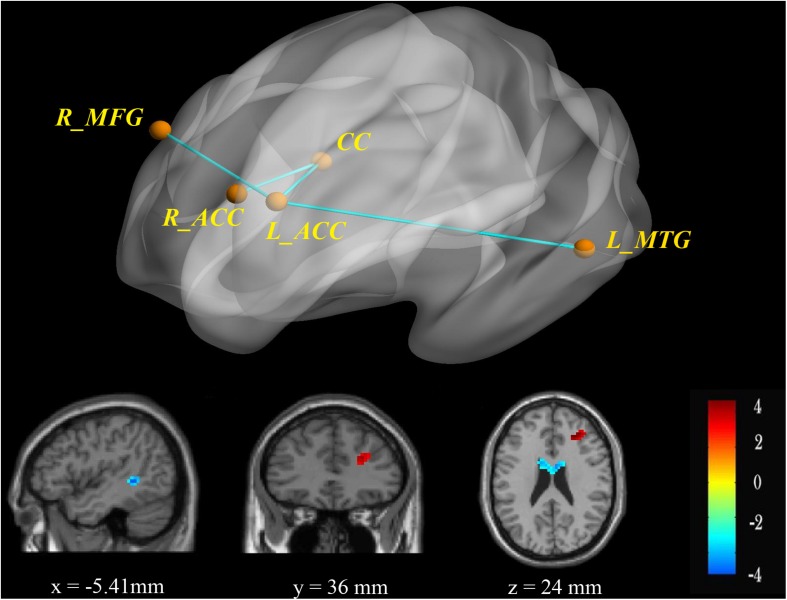
RsFC of the bilateral ACC as seeds. There was increased (red) rsFC between the left ACC and right MFG, as well as decreased (blue) rsFC between the left ACC and left MTG in the BPD group compared with the HC group. FWE-corrected *p* < 0.05 significance criterion. Color bar indicates the *T-*score.

Borderline personality disorder patients exhibited significantly increased rsFC of the ACC with the right MFG and decreased rsFC with the left MTG compared with HCs. In addition, the BPD group had lower connectivities between both ACC sides and the corpus collosum (CC) than HCs (Table 2 and Figure 1).

**TABLE 2:** Significant differences in FC between BPD patients and HCs.

**Cluster**	***P*__FWE–corr_**	**Brain region/**	**Voxels**	***t***	**MNI**
		**fasciculus**			**coordinates**
					**(X, *Y, Z*)**
**ACC_L as a seed**					
FC↑ Cluster1	0.041	Frontal_Mid_R	42	4.85	27, 36, 24
FC↓ Cluster2	0.037	Temporal_Mid_L	43	–5.41	−45, −45, 0
FC↓ Cluster3	0.026	Corpus callosum	47	–4.85	6, 3, 24
**ACC_R as a seed**					
FC↓ Cluster1	0.013	Corpus callosum	56	–5.13	6, 3, 24

*BPD, borderline personality disorder; HC, healthy controls; ACC_L, left anterior cingulate cortex; ACC_R, right anterior cingulate cortex; FC, functional connectivity; Frontal_Mid_R, right frontal middle gyrus; Temporal_Mid_L, left temporal middle gyrus.*

### SC

**FIGURE 2:**
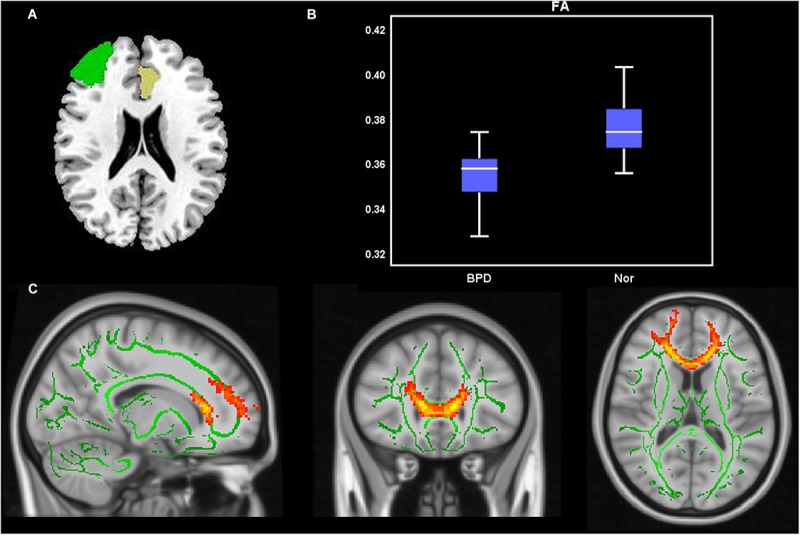
Probabilistic fiber tracking from the left ACC to the right MFG. **(A)** The seed (left ACC, yellow) and target (right MFG, green) were based on the AAL90 template and back-projected to the individual’s native space in sagittal, coronal, and axial planes. **(B)** Group comparisons of FA and MD values for fiber bundles passing though the left ACC to the right MFG (*p* < 0.05). *Y*-axis presents the mean of FA and MD values. The error bars present the standard error of mean. **(C)** Fiber distribution: fibers starting from the left ACC passed through the CC and the right ACC and then entered the right MFG.

Compared with HCs, the BPD group had significantly decreased FA values in left ACC-to-right MFG tract (Table 3 and Figure 2), findings suggestive of impaired fiber integrity. The tract probability of left ACC-to-right MFG and left ACC-to-left MTG tract did not differ between the two groups.

**TABLE 3:** Comparison of probabilistic fiber tracking between BPD patients and HCs.

**Seed to target**	**Fiber metric**	**BPD group (*N* = 46)**	**HC group (*N* = 44)**	***F***	***P***
ACC_L to Frontal_mid_R	FA	0.35 ± 0.10	0.37 ± 0.02	–7.85	<0.01
	MD (×10^–3^)	0.89 ± 0.03	0.91 ± 0.05	–3.04	0.08
	Probability (×10^–1^)	0.11 ± 0.16	0.09 ± 0.11	0.23	0.6
ACC_L to Temporal_mid_L	FA	0.31 ± 0.01	0.32 ± 0.01	0.11	0.74
	MD (×10^–3^)	0.81 ± 0.02	0.81 ± 0.02	–2.63	0.11
	Probability (×10^–2^)	0.02 ± 0.42	0.02 ± 0.06	–1.52	0.22

*BPD, borderline personality disorder; HC, healthy controls; ACC_L, left anterior cingulate cortex; Frontal_Mid_R, right frontal middle gyrus; Temporal_Mid_L, left temporal middle gyrus; FA, fractional anisotropy; MD, mean diffusivity.*

### Associations Between Imaging Parameters and Psychological Variables in BPD Patients

Pearson correlation analyses showed that CERQ-negative subscale scores correlated negatively with rsFC between the left ACC and CC (*r* = −0.59, *P* = 0.032), and CES-D scores correlated negatively with rsFC between the left ACC and right MFG (*r* = −0.37, *P* = 0.037) after multiple comparisons with Bonferroni correction. There were no other significant correlations between psychological variables (negative affective SAIS or STAI) and imaging parameters (rsFC values, tract probability, mean FA, or MD).

## Discussion

In the current study, combining rsFC and probabilistic fiber tracking with the bilateral ACC as seeds, we explored the SC and FC of the ACC and examined correlations between abnormal neuroimaging indices and psychological variables in BPD patients. We found that patients with BPD exhibited abnormal SC and FC in the emotion-regulation-related ACC-CC-frontal neural circuit. These findings suggest that abnormal SC and FC in frontal–limbic structures of the brain could mediate emotional dysregulation in patients with BPD.

### SC and FC Between the Left ACC and Right MFG

The ACC and PFC are key brain regions in emotional regulation and impulsiveness (Davidson and Irwin, 1999). Previous on-task fMRI studies utilizing emotion regulation strategies (e.g., restraint and reappraisal) have suggested that the ACC and other frontal regions (i.e., orbital frontal cortex, dorsolateral PFC, dorsomedial PFC, and ventrolateral PFC) are involved in emotional regulation processing (Beauregard et al., 2001; Lévesque et al., 2003; Ochsner et al., 2004; Phan et al., 2005; Urry et al., 2006). More specifically, the MFG, a component of the dorsolateral PFC, is particularly important in mood regulation and suppression of unwanted memories (Kluetsch et al., 2012; Krause-Utz et al., 2014a). In the current study, we found increased left ACC-to-right MFG FC in patients with BPD, consistent with previous findings (Krause-Utz et al., 2012). Based on rsFC data, we plotted the left ACC-to-right MFG fiber bundle in BPD patients using probabilistic fiber tracking and found that left ACC efferents passed through the CC and projected to the contralateral MFG, consistent with normal anatomy (Rüsch et al., 2010b). Importantly, we found that WM integrity from the left ACC to the right MFG was impaired with decreased FA in the BPD group compared with the HC group. The presently observed altered rsFC and impaired WM fasciculus integrity between the ACC and MFG suggest that emotional regulation circuitry may be deficient in BPD patients.

### FC Between the Left ACC and Left MTG

Alterations in the brain default model network (DMN), including the medial PFC, ACC, cuneus/precuneus, MTG, thalamus, and insular cortex, which are located mainly along the midline of the brain, were found in a prior rs-fMRI study in BPD patients (Wolf et al., 2011). The ACC and MTG are important for numerous important DMN functions, including: separating internal cognition from external stimulus processing; autobiographical memory processing; and monitoring of cognitive, emotional, and somatosensory states (Buckner et al., 2008; Spreng et al., 2009; Napadow et al., 2010). In a previous rs-fMRI study, we found decreased connectivity–amplitude coupling in the left MTG, suggesting that the left MTG may be a functionally impaired hub in BPD (Lei et al., 2018). The MTG also has decreased rs activation (Wingenfeld et al., 2009) and increased activity during emotional processing in BPD patients (Guitart-Masip et al., 2009). Together with previous studies (Wolf et al., 2011; Lei et al., 2017), our study found decreased rsFC between the left ACC and the left MTG in BPD patients, which suggested disturbed DMN and emotional processing in patients with BPD.

### SC and FC Between the ACC and CC

The CC mediates communication between the hemispheres (Luders et al., 2010). The WM integrity of the CC genu and body has previously been shown to be impaired in BPD patients (Salvador et al., 2016). Moreover, the CC isthmus was found to be thinner in BPD patients than in HCs, suggesting that inter-hemispheric SC may be affected in BPD (Rüsch et al., 2007). The present finding of decreased rsFC between the bilateral ACC and the CC is consistent with the possibility that SC between the cerebral hemispheres may be abnormal in BPD patients. Because the ACC is involved in regulating emotion and behavior (Minzenberg et al., 2007; Silbersweig et al., 2007; Koenigsberg et al., 2009; Niedtfeld et al., 2012; Perez et al., 2016; Schulze et al., 2016), decreased interhemispheric connectivity between the left and right ACCs may be a pathological correlate of BPD.

### Psychometric Correlates of ACC Abnormalities in BPD

Patients with BPD have been shown consistently to be hypersensitive to negative emotional stimuli, with emotional dysregulation and negative regulatory strategies that are often accompanied by anxiety and depression (Yen et al., 2002; Niedtfeld et al., 2010; Schulze et al., 2016; Yang et al., 2018). The present findings of significantly higher SAIS-negative affectivity, CERQ-negative subscale, CES-D, and STAI scores in BPD patients than HCs are consistent with heightened negative intensity, depression, and anxiety levels as well as reliance on negative emotion cognition regulation strategies in BPD.

A frontal–limbic system suppression model in which there is reduced PFC control of hyperactive limbic areas has emerged as a favored hypothesis of the neurological basis of emotional dysregulation in BPD (Agrawal et al., 2004). More specifically, amygdalar hyperactivity with hypoactivity in the ACC and PFC (medial, orbital, and dorsolateral) is typical during negative emotional processes in BPD (Silbersweig et al., 2007; Perez et al., 2016; Baczkowski et al., 2017). In this study, cognitive emotion regulation scores correlated negatively with left ACC-CC rsFC, while depressive scores correlated negatively with left ACC–right MFG rsFC. These results provide further support for the view that emotional regulation circuitry may be impaired in BPD patients.

### Limitations

Although we combined rsFC and SC analyses to investigate links between function and structure in BPD, which had not been thoroughly assessed before, this study had some limitations. Firstly, FC was assessed in a rs rather than in an on-task state. We are planning future studies that will focus on FC during the performance of emotion-related tasks. Secondly, because this study focused primarily on the emotion-related ACC and associated frontal regions, connectivities among other emotion-related limbic structures (e.g., amygdala) remain to be investigated further to provide a more comprehensive understanding of brain connectivity in BPD.

## Conclusion

In this study, using rs-fMRI and probabilistic fiber tracking, we combined SC and FC data to investigate BPD alterations in ACC-based circuitry. With the left ACC selected as a seed, BPD patients exhibited increased rsFC and abnormal SC with the right MFG, and decreased rsFC with the left MTG, compared with HCs. Further, rsFC of the left ACC correlated negatively with negative cognitive emotion regulation and depressive symptoms in BPD patients. Given that the ACC plays an important role in emotional cognitive control, abnormal connectivity of the ACC supports the possibility that BPD may be characterized by deficient emotional regulation circuitry. Collectively, our data suggest that connectivity of these brain regions may be important imaging biomarkers in BPD populations, which could have clinical implications for treatment of the disorder.

## Ethics Statement

This study was approved by the ethics committee of the Central South University, and each participant signed an informed consent form at enrollment.

## Author Contributions

XL analyzed the data and wrote the manuscript. BZ, HY, WP, QL, and YZ collected the data. MZ, CT, and JY gave the idea and revised the manuscript. SY also helped the manuscript in revision.

## Conflict of Interest Statement

The authors declare that the research was conducted in the absence of any commercial or financial relationships that could be construed as a potential conflict of interest.

## Footnotes

1 http://rfmri.org/DPARSF

2 http://www.fil.ion.ucl.ac.uk/spm/

3 https://fsl.fmrib.ox.ac.uk/fsl/fslwiki/FSL

## Abbreviations

ACC: anterior cingulate cortex
BPD: borderline personality disorder
CC: corpus collosum
DTI: diffusion tensor imaging
FA: fractional anisotropy
FC: functional connectivity
HCs: healthy controls
MD: mean diffusivity
MFG: middle frontal gyrus
MTG: middle temporal gyrus
PFC: prefrontal cortex
rs-fMRI: resting state functional magnetic resonance imaging
SC: structural connectivity
WM: white matter.
